# A Split from Traditional Orbital Compartment Syndrome Intervention: Case Report of Vision-saving Vertical Lid Split Procedure

**DOI:** 10.5811/cpcem.52953

**Published:** 2026-05-23

**Authors:** Hannah Chason, Barret Zimmerman, Andrew Jenzer, David Fay, Julia Elpers

**Affiliations:** * Brown University, Department of Emergency Medicine, Providence, Rhode Island; † Harvard Medical School and Mass General Brigham, Department of Emergency Medicine, Boston, Massachusetts; ‡ Uniformed Services University of Health Sciences, Womack Army Medical Center, Department of Surgery, Oral and Maxillofacial Surgery Residency, Fort Bragg, North Carolina; § Midwest Eye Center, Department of Opthomalmology, Cincinnati, Ohio

**Keywords:** trauma, ophthalmology, procedures, vertical lid split, orbital compartment syndrome, case report

## Abstract

**Introduction:**

Many emergency physicians will never perform a lateral canthotomy and cantholysis, and one-third of those who try will be unsuccessful at relieving the pressure that threatens permanent vision loss. This procedure is notoriously difficult and rare, but a recently proposed alternative—the vertical lid split—may be simpler and more effective.

**Case Report:**

We report the case of a 35-year-old woman with motor vehicle collision-related orbital trauma who presented to a community emergency department. Initially, she had intact vision and extraocular movements. Imaging showed a comminuted inferior orbital blowout fracture with retrobulbar hemorrhage, and the transfer process was initiated. However, after coughing she developed vision loss and elevated intraocular pressure. Lateral canthotomy and cantholysis was performed for suspected orbital compartment syndrome but did not fully address the elevated pressures or restore vision. The emergency physician then performed a vertical lid split procedure, which fully restored vision and normalized pressures.

**Conclusion:**

To the best of our knowledge, this is the first case report of orbital compartment syndrome to be treated with vertical lid split, and the first case report of any full-thickness eyelid incision technique being used for salvage of an unsuccessful lateral canthotomy and cantholysis; the result was excellent.

## INTRODUCTION

Orbital compartment syndrome is a rare (about 1:1,000 facial trauma cases), vision-threatening emergency, driven by accumulation of any substance within the rigid confines of the orbit.^1^,^2^ Most emergency department (ED) cases are due to trauma, and most of those are secondary to retrobulbar hematoma; other causes include air, edema, pus, tumors, or foreign bodies. Increasing pressure directly injures neurosensory structures and causes optic nerve and retinal ischemia via limitation of arterial and venous flow. This progresses to irreversible vision loss within as little as one hour.^3^ It is a clinical diagnosis made from history, exam, and testing, including tonometry.

Exam findings include proptosis, severe pain, a “hard eye,” ophthalmoplegia, abnormal pupillary light reflex, and relative afferent pupillary defect. There is progressive loss of color saturation, visual acuity and, finally, all light perception.^1^,^4^ Intervention should be considered when pressures enter the 30–40 millimeters of mercury (mm Hg) range (reference range: 8–21 mm Hg). While pressure is one of the most useful metrics in guiding surgical decompression, clinical judgment is the ultimate factor.^1^,^4^,^5^ The most widely known intervention for treatment of orbital compartment syndrome is lateral canthotomy and cantholysis. Most (66%) emergent lateral canthotomy and cantholysis procedures for orbital compartment syndrome are performed by emergency physicians, rather than ophthalmologists; however, one-third of those attempts at relieving pressure using this technique in the ED are unsuccessful.^6^

Our multidisciplinary team of authors (military facial trauma surgeon, oculoplastic surgeon, emergency attending, emergency medicine (EM)/critical care fellow, and EM resident), have experienced that lateral canthotomy and cantholysis is challenging to perform in an edematous trauma patient with distorted anatomy using disposable tools. In our experience, this has been true for Special Operations Forces medics, experienced emergency physicians, and even ophthalmologists. Furthermore, even full cantholysis may still be inadequate at relieving sufficient pressure to prevent poor outcomes. More than six surgical interventions have been proposed for orbital compartment syndrome, one of which is gaining interest in the EM community.^6^–^9^

At a 2017 ophthalmic plastic and reconstructive surgery symposium, Dr. Roxana Fu proposed that the “vertical lid split” procedure (already used by oculoplastics surgeons) could be used for acute orbital compartment syndrome. In 2021 Drs. Fu and Elpers published a test of this hypothesis in cadavers with good results.^7^ That same year, 85% of surveyed ophthalmologists and emergency physicians reported that they had never heard of the vertical lid split for orbital compartment syndrome. And even after being educated on the technique, 83% said they would still choose to perform lateral canthotomy and cantholysis first.^10^

To our knowledge, this is the first case report of the vertical lid split being used on a patient, and the first case report of any full-thickness eyelid incision technique being used to salvage a failed lateral canthotomy and cantholysis. Despite the skepticism expressed by ophthalmologists and emergency physicians in the 2021 survey, the case we describe is possibly the scenario in which many emergency clinicians could use this technique.

## CASE REPORT

A 35-year-old female presented to our community ED with isolated left orbital trauma after she struck her face during a motor vehicle collision. She had brief amnesia to the event but was fully oriented and neurologically intact. She had bruising, swelling, and a small laceration to her forehead and left upper eyelid. Vision, extraocular movements, and pupillary exam were normal. There was no fluorescein uptake or Seidel sign.

Swelling and proptosis improved with ice. Computed tomography of the brain, face, and cervical spine demonstrated a left-sided comminuted inferior orbital blowout fracture with extensive intra/extraconal gas and blood, as well as retrobulbar hemorrhage contributing to significant proptosis of the eye. The extraocular muscles were superiorly displaced without appreciable herniation through the fracture. The globe was intact. Intraocular pressure was then checked and found to be 36 mm Hg (left) and 10 mm Hg (right).

*CPC-EM Capsule*

What do we already know about this clinical entity?

*Orbital compartment syndrome is a vision-threatening emergency. Lateral canthotomy with cantholysis has been the primary intervention.*

What makes this presentation of disease reportable?

*Successful decompression was obtained using a vertical lid split procedure, following failed lateral canthotomy with cantholysis.*

What is the major learning point?

*The vertical lid split is an effective, potentially faster, and easier treatment for orbital compartment syndrome. It can also be used as a salvage technique.*

How might this improve emergency medicine practice?

*Knowledge of the vertical lid split could lead to improved success in treating orbital compartment syndrome.*

Lateral canthotomy and cantholysis was considered but deferred as the patient had normal vision, minimal pain, improving proptosis, and intraocular pressure < 40 mm Hg. The lacerations were repaired, and transfer to a trauma center was initiated for facial surgery and ophthalmology. In the 30 minutes that elapsed while awaiting transfer, the patient vomited. She immediately complained of severe left eye pain, and we noted increased swelling and proptosis. She was no longer able to open her left eye secondary to the swelling, and when her lids were manually retracted, she reported complete loss of light perception.

Lateral canthotomy and cantholysis was attempted by the third-year EM resident and attending. The degree of proptosis and swelling made visualization of the canthal ligaments challenging. After cutting what we believed were the inferior and superior canthal ligaments, the patient did have transient improvement in her vision (able to see lights and shapes), but shortly thereafter she lost it again completely.

A vertical lid slit was then performed (Image). Both lids were anesthetized with lidocaine containing epinephrine. Using the scissors from a disposable laceration repair kit, a full-thickness 8-mm incision was made vertically through the upper eyelid, 4 mm from the lateral canthus. Vision was not restored; thus, another 7 mm cut was made to the lower lid, 4 mm from the lateral canthus. The patient had improvement in her vision within 30 seconds. At this time the transport team arrived, and she was transported.

On arrival to the trauma center, she was evaluated by ophthalmologists, who found her visual acuity to be 20/400 (left eye), 20/20 (right eye), and intraocular pressures 24 mm Hg (left eye) and 22 mm Hg (right eye). She was admitted for nausea and pain control. Upon discharge, her left eye pressure was 21 mm Hg, and her visual acuity was 20/20. She was given ophthalmology follow-up for lid repair and plastic surgery follow-up for her fractures.

## DISCUSSION

To our knowledge, this is the first published case of the vertical lid split being performed in an emergency setting, and in this case the patient went from no light perception to a full restoration of her normal vision. Prior to attempting the vertical lid split, the experienced EM team (who had performed multiple lateral canthotomy and cantholysis procedures on living patients, as well as procedures on cadaveric models) attempted the current “standard,” achieving only partial improvement in pressures and only transient improvement in vision.

Vertical lid split, a procedure that involves lysis of the upper and lower eyelids, has traditionally been used by ophthalmology for increased access to the orbit. Subsequent cadaveric evidence demonstrated that it could be used to address orbital compartment syndrome and that the lysis of both eyelids (vertical lid split) was superior to isolated lysis of the lower lid (“one-snip”), while providing an additional 10 mm Hg-reduction in intraocular pressures.^9^,^11^–^13^ To perform the technique, local anesthesia is infiltrated and full-thickness, vertical incisions are made through both upper and lower eyelids with scissors to completely transect the tarsal plates (8–10 mm for the upper eyelid and 7–8 mm for the lower eyelid) approximately 4 mm from the lateral canthus (Figure).

Incised layers include skin, orbicularis oculi muscle, the dense tarsal plate, and conjunctiva. Incisions here are relatively safe and easily repaired by a surgical subspecialist with good cosmetic outcomes. In our case, the ophthalmologists at the trauma center were impressed with the technique and were not critical of this approach.

Based on our collective experience with hundreds of lateral canthotomy and cantholysis procedures and vertical lid splits performed or repaired, and > 1,000 trauma resuscitations in the ED, we believe the vertical lid split is faster, simpler, and more reliable in the emergency setting than lateral canthotomy and cantholysis. Furthermore, a cadaveric study showed it was non-inferior at pressure reduction.^7^ “Time is vision” in orbital compartment syndrome, and these patients may have other time-critical pathology; a more efficient procedure allows the team’s attention to shift more quickly to the patient’s other treatment priorities.

In our experience, we have seen emergent lateral canthotomy and cantholysis performed by emergency physicians, ophthalmologists, and oral and maxillofacial surgeons. The procedures take over 10 minutes, requiring multiple cuts, rechecking pressure, more cuts, and often result in failure. Sometimes, with experienced clinicians and more ideal anatomy, lateral canthotomy and cantholysis can be less protracted, but for many emergency physicians this is a once-in-a-career procedure. A final advantage is that a fast, simple, and more intuitive procedure may increase clinician comfort and the likelihood of *actually* initiating the procedure. A British survey showed that many emergency clinicians would prefer to pursue other testing or delay intervention for specialist involvement rather than attempting to perform lateral canthotomy and cantholysis, citing lack of knowledge and comfort in performing the procedure.^14^

Disadvantages of the vertical lid split are similar to those of lateral canthotomy and cantholysis. Incomplete cuts can fail to relieve the pressure, and an errant instrument could damage the globe. Postprocedural complication would mostly be caused by failure to follow-up for subspecialist repair in a timely manner (5–7 days). Complications include entropion, ectropion, cosmetic defects, scarring, and potential need for revision surgery. Finally, given how entrenched the lateral canthotomy and cantholysis technique is in EM education, clinicians may still be hesitant to perform the vertical lid split. Hopefully, this case will help clinicians feel more comfortable with this technique as either a primary or salvage procedure.

## CONCLUSION

This is the first documented case of the vertical lid split being used for orbital compartment syndrome. The speed and ease of the procedure and successful restoration of vision after an unsuccessful lateral canthotomy and cantholysis reinforce its role in an emergency physician’s skillset. Treating orbital compartment syndrome may be a once-in-a-career experience for many emergency physicians, and the evidence shows there is room for improvement in our current approach. This case may represent a potential paradigm shift in the treatment of orbital compartment syndrome.

## Figures and Tables

**Figure:**
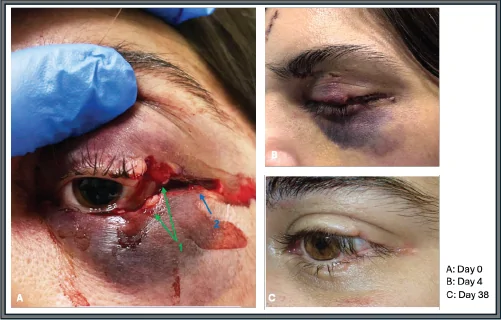
Location of incisions for vertical lid split procedure and lateral canthotomy and cantholysis.

**Image.**
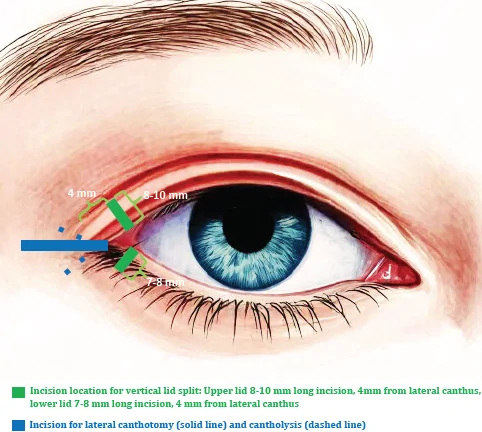
The upper- and lower-lid incisions from the vertical lid split can be appreciated (1: green arrows), as well as the lateral canthotomy and cantholysis incision (2: blue arrow). Image A was obtained after the patient arrived at the trauma center, and swelling was significantly decreased. Images B and C were obtained on a follow-up visit with ophthalmology on days 4 and 38 after the procedure, before surgical repair.
